# Long Non-coding RNA *TDRKH-AS1* Promotes Colorectal Cancer Cell Proliferation and Invasion Through the β-Catenin Activated *Wnt* Signaling Pathway

**DOI:** 10.3389/fonc.2020.00639

**Published:** 2020-05-15

**Authors:** Yang Jiao, Jialiang Zhou, Yecheng Jin, Yingxin Yang, Mingxu Song, Ling Zhang, Jiayan Zhou, Jiwei Zhang

**Affiliations:** ^1^College of Life Sciences and Oceanography, Shenzhen University, Shenzhen, China; ^2^The Affiliated Hospital of Jiangnan University, Wuxi, China; ^3^Affiliated Sir Run Run Shaw Hospital, Zhejiang University School of Medicine, Hangzhou, China; ^4^College of Life Science, Zhejiang Chinese Medical University, Hangzhou, China; ^5^Department of Veterinary and Biomedical Sciences, College of Agricultural Sciences, The Pennsylvania State University, University Park, PA, United States; ^6^Department of Statistics, Eberly College of Science, The Pennsylvania State University, University Park, PA, United States; ^7^The MOE Key Laboratory for Standardization of Chinese Medicines, Institute of Chinese Materia Medica, Shanghai University of Traditional Chinese Medicine, Shanghai, China

**Keywords:** colorectal cancer, *TDRKH-AS1*, *Wnt* signal pathway, β-catenin, TCGA, RNA sequencing analysis, differential gene expression

## Abstract

Colorectal cancer (CRC) is a common cancer worldwide, with a lower 5-years survival rate. Recently, long non-coding RNAs (lncRNAs) have been well-studied as the oncogenes or the tumor suppressors in multiple malignancies, including CRC. However, their biological functions and potential mechanisms in human cancer remain unclear. Here, we evaluated the expression of *TDRKH-AS1* in CRC tissues and identified its potential targets. We found that *TDRKH-AS1* is upregulated in majority of CRC patients, which is also significantly correlated with their malignant characteristics and their dismal prognoses. The high expression of *TDRKH-AS1* can promote cancer cell proliferation substantially and invasion based on *in vitro* experiments. We also recognized that the *TDRKH-AS1* targets the β-catenin in the *Wnt* signaling pathway to exert its carcinogenic activity. *TDRKH-AS1* could serve as a promising prognostic predictor and a potential therapeutic target for further early diagnoses and treatments via a non-invasive method.

## Introduction

Colorectal carcinoma (CRC) is one of the most common malignancies with high mortality rates in the world, especially in the United States and European countries since last century (1, 2). According to the estimation of the International Agency for Research on Cancer (IARC), the number of incident cases in 2030 will reach 1.5 million, a 38% increase from 2018. Nowadays, more CRC cases were identified outside the western countries. In China, the morbidity and mortality associated with CRC are steadily increasing (3). Currently, the invasive treatments and chemotherapies are widely used for rescuing the CRC patients, including the endoscopic or segmental resection for malignant polyp, resection with adjuvant chemotherapy for cancer with a high potential of metastasis, and the chemotherapy, immunotherapy, or radiotherapy for cancer with metastasis to the adjacent tissues (4, 5). However, patients usually suffer from side effects of surgery and chemotherapy, such as pain, internal bleeding, and infection. Therefore, it needs to study the incidence, development, and prognosis of colorectal cancer to find a new theoretical basis for the non-invasive personalized treatment of colorectal cancer.

Long non-coding RNA (LncRNA) is a gene fragment that is more than 200 nucleotides and has no or weak possibility to be utilized for producing proteins (6). The expression of lncRNA accounts for 90% of all expressed RNAs in human. Although the lncRNA does not code for proteins, it plays a significant role in chromatin modification, transcriptional regulation, and post-transcriptional regulation (7). In the past studies, the lncRNAs were well-studied for understanding their critical roles in the development of cancers (8–10). Recently, multiple lncRNAs were identified in the regulation of chromosomes under the pathological conditions of cancer (11, 12). The association between the alteration of *TDRKH-AS1* copy number and the survival rates of lung adenocarcinoma patients was reported in a high-throughput computational study (13). *TDRKH-AS1* is a lncRNA with three spliceosomes with the chromosomal location of 1q21.3. As an antisense RNA, although *TDRKH-AS1* always plays a crucial role in tumor development, it has not been studied in detail.

Here, we located the upregulated *TDRKH-AS1* in CRC patients and carried out its biological function in the *Wnt* signaling pathway based on the patient data from “The Cancer Genome Atlas” (TCGA) database. TCGA is a government-funded project that provides publicly available data sets to help improve diagnostic methods, treatment standards, and ultimately cancer prevention from major cancer-causing genomes. The *Wnt* signaling pathway is a set of signal transduction pathways with multiple downstream channels stimulated by the binding of the ligand-protein *Wnt* and the membrane protein receptor. The extracellular signals are required to be transmitted into the cell through the activation of intracellular receptors on the cell surface (14). The overactivation of the *Wnt* pathway was previously identified as the most critical driver of CRC (15). We further used the multiple biological assays to prove that the *TDRKH-AS1* could regulate the gene and protein expressions and promote cancer proliferation and metastasis. Therefore, the highly expressed *TDRKH-AS1* is related to clinical prognosis for CRC patients. It can be used as a novel molecular marker with a non-invasive potential for the diagnosis and treatment of colorectal cancer.

## Materials and Methods

### Cell Lines and Culture

HCT-116 and LoVo cells were received from the Chinese Academy of Sciences cell bank. Cells were thawed and cultured in RPMI1640 medium [10% fetal bovine serum (FBS)] at 37°C with a concentration of CO_2_ of 5%.

### Cell Counting Kit-8

The cell counting kit-8 (CCK-8) assay (Dojindo, Kyushu, Japan) was used for evaluating the cell proliferation in the LoVo cells and HCT-116 cell lines by following the instructions. Approximately 1,000 cells per well were seeded into three wells on a 96-well plate, and 10 μl of CCK-8 was added to each well after being cultured for 24, 48, and 72 h. The cells were then analyzed for absorbance [optical density (OD) value] at 450 nm.

### Transwell Assay

The cell suspension of the LoVo cells transfected with *siTDRKH-AS1* and the HCT-116 cells transfected with *TDRKH-AS1* were added to the upper chamber containing RPMI1640 culture medium, and the lower chamber was filled with a cell suspension with 10% FBS. The LoVo cells transfected with negative control small interfering RNA (siRNA) (siNC, RiboBio, Guangzhou, China) and MOCK (phosphate-buffered saline, PBS), and the HCT-116 cells transfected with an empty vector (pcDNA3.1, Addgene, Watertown, MA, USA) were used as the negative controls for each cell line. The LoVo cells transfected with an empty vector (pcDNA3.1) carrying *siHOTAIR* or *siFEZF1-AS1* and the HCT-116 cells transfected with an empty vector (pcDNA3.1) carrying *HOTAIR* or *FEZF1-AS1* were used as the positive controls for cell-line-based Transwell assay experiments (16, 17). Finally, the migrated cells were fixed by using 4% paraformaldehyde and stained with crystal violet.

### Quantitative Real-Time Polymerase Chain Reaction

The TRIzol Reagent (Invitrogen, Carlsbad, CA, USA) was used to extract total RNA from the cell lines. The complementary DNA (cDNA) library was obtained from the reverse transcription using the reverse transcriptase kit (Takara Bio, Dalian, China). The 10-μl system was placed in a 384-well plate for the quantitative real-time polymerase chain reaction inside the LifePro Thermal Cycler (Hangzhou Bioer Technology, Hangzhou, China). The RNA level was monitored by 7900 Real-Time PCR System with the SDS v.2.3 software sequence detection system (Applied Biosystems, Waltham, MA, USA) using the SYBR Green (Takara Bio, Dalian, China) as reference. β-Actin served as the internal reference for *TDRKH-AS1*, and the results were analyzed by the 2–ΔΔCt method (18).

### Luciferase Activity Assay

Cells were cotransfected with β-catenin luciferase reporter constructs based on the backbone of luciferase plasmid (pBV-Luc, Addgene, Watertown, MA, USA) and Renilla plasmid (pcDNA3.1-ccdB-Renilla, Addgene, Watertown, MA, USA) according to the manual instruction. Cell lysis and luciferase activity were measured using the Luc-Pair™ Dual-Luminescence Assay Kit 2.0 (GeneCopoeia, Rockville, MD, USA) according to the manufacturer's instructions.

### Flow Cytometry Assay

Logarithmic CRC cells were adjusted to 3 × 10^5^ cells/ml and seeded on six-well plates (2 ml/ml). After cell adhesion, the cells were transfected with the *TDRKH-AS1* interference sequence or the control sequence. After 48 h of culture, two groups of cells were collected and washed with PBS. Furthermore, cells were blocked using 2% FBS for 30 min and then washed with PBS. The cells were then dyed and placed in the dark for 30 min. The apoptosis rate of each group was determined by flow cytometry. Cells were treated and collected in a similar manner, and then, the cells were precooled with 75% ethanol and fixed in a refrigerator at −20°C overnight. The content of DNA was detected by propidium iodide (PI) staining, and the proportion of each phase was calculated by the software.

### Statistical Analysis

Statistical analysis was carried out using GraphPad Prism (version 8.2.1) software. Two-way *t*-test and receiver operating characteristic curve analysis were used for analyzing the differences between the two groups. All the assays were repeated at least three times. Statistics were considered to be at a significant level when *p* < 0.05, where ^*^p < 0.05, ^**^p < 0.01, and ^***^p < 0.001.

### Gene Set Enrichment Analysis

To identify the relevant pathways that were regulated by *TDRKH-AS1*, the correlation coefficient (*R*) was calculated between *TDRKH-AS1* and other genes in the CRC dataset from TCGA. Further, genes were ranked by their coefficients. The enrichment score (ES) for each pathway was calculated, and the significant level of the ES was also estimated from gene set enrichment analysis (GSEA) (19, 20). Then, the proportion of leading genes for each significantly enriched pathway was calculated with false discovery rate (FDR) <1% from GSEA. Only the pathways related to the cancer hallmark gene sets from MsigDB were considered (21).

### Differential Gene Expression Analysis

The RNA-sequencing data of 71 CRC patients' tumor tissues and adjacent non-tumor tissues were obtained from TCGA (https://gdc.cancer.gov/) database. The individual demographic information and disease progress (stage, disease type, primary site, and vital status) were extracted and sorted from TCGA (Supplement 1). The differential gene expression analysis was conducted for comparing the later stages (stage II, IIA, III, IIIB, IIIC, and IV) and initial stage I using the *limma* package in R software (Supplement 2) (22). Genes with a count per million (CPM) above 0.5 in at least two cell lines were selected. *Voom* transformation was applied for implementing the normalization factors (23). The Empirical Bayes Rule was also implemented to shrink the variance. The *t* statistics and the *p*-values were estimated using the *eBays* function within the *limma* package in R software. Genes were annotated based on *org.Hs.eg.db* package in R software (24). Gene functional enrichment tests for biological processes, cellular components, and molecular functions were performed based on the gene ontology (GO) terms.

## Results

### *TDRKH-AS1* Is a Long Non-coding Antisense RNA That Will Promote the Progression of CRC

**Figure 1:**
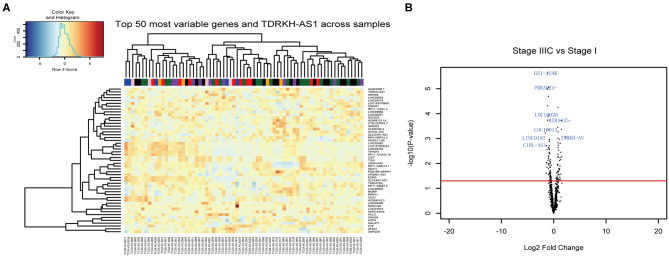
*TDRKH-AS1* is a long non-coding antisense RNA that is upregulated in colorectal cancer (CRC). **(A)** The 71 CRC tissue samples in The Cancer Genome Atlas (TCGA) database were analyzed and sorted into stage I (black), stage II (blue), stage IIA (green), stage III (orange), stage IIB (purple), stage IIIC (pink), and stage IV (red). **(B)** The long non-coding RNAs (lncRNAs) with high abundance were verified in the CRC TCGA database.

The lncRNAs have a critical role in cancer progresses. In this study, we identified the *TDRKH-AS1* using the 71 CRC patients' RNA sequencing data from TCGA database. The fluctuation in the expression of the RNAs was focused on CRC between the patients in later stages with the initial stage (Figure 1A). Although the expression of the RNAs varied from stage to stage, the *TDRKH-AS1* was highly overexpressed in two patients in stage IIIC. The *TDRKH-AS1* was also identified as one of the top hits when comparing the gene expression between the patients in stage IIIC and patients in stage I (Figure 1B). We further processed the gene enrichment analyses in biological processes (Supplement 3), molecular functions (Supplement 4), and cellular components (Supplement 5) between the stage IIIC and stage I CRC patients. The differential expression of genes in patients would lead to the differences in the functions of negative regulations of the gene silencing, DNA and RNA polymerase activities, and messenger RNA (mRNA) and regulatory RNA bindings.

### *TDRKH-AS1* Is Upregulated in CRC and Has a Positive Correlation With CRC Prognosis

**Figure 2:**
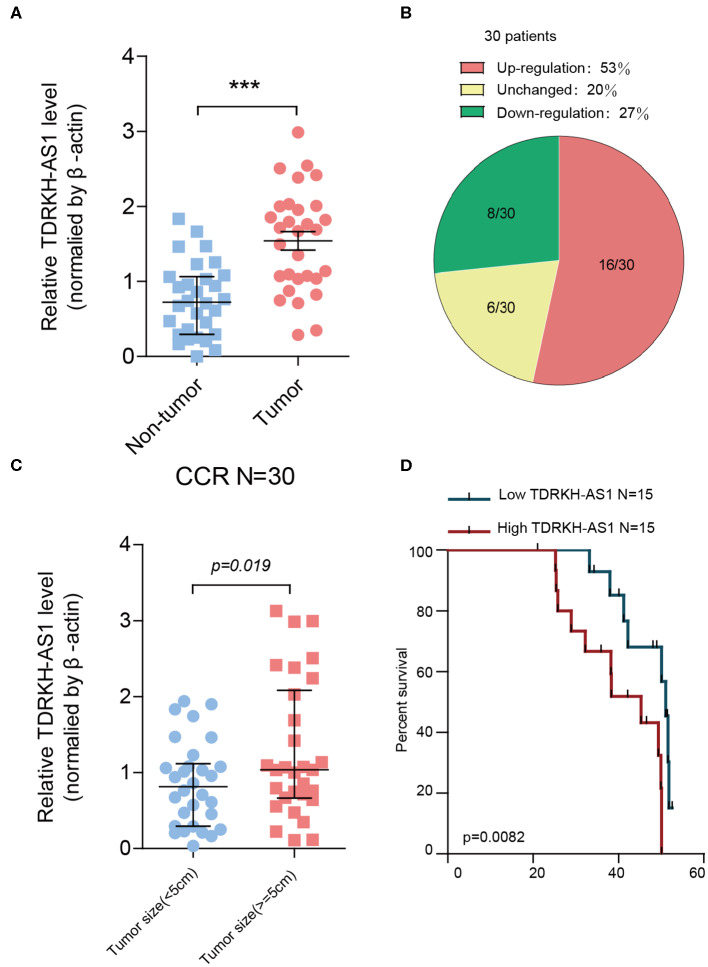
*TDRKH-AS1* is upregulated in colorectal cancer (CRC) and negatively correlated with the clinical outcomes of patients of CRC. **(A)** The relative *TDRKH-AS1* RNA levels were quantified in 30 pairs of CRC tissues and adjacent normal tissues by quantitative real-time PCR (qRT-PCR). **(B)** The pie chart indicates that the ratio of 30 tumor sample *TDRKH-AS1* levels in the upregulation (red), unchanged (yellow), and downregulation (green) categories. **(C)** The upregulation of *TDRKH-AS1* is positively correlated with the tumor size (≥5 cm). **(D)** Using Kaplan–Meier to analyze the relationship between *TDRKH-AS1* levels and the percent survival in 30 CRC patients. Statistics were considered to be at a significant level when *p* < 0.05, where ^*^p < 0.05, ^**^p < 0.01, and ^***^p < 0.001.

From the gene differential expression analyses, we figured out that *TDRKH-AS1* was an upregulated lncRNA in TCGA database of CRC. Therefore, we further testified the expression of 30 pairs of CRC patients' tumor tissues and adjacent non-tumor tissues in the laboratory. We first analyzed the relationships between *TDRKH-AS1* expression and clinical–pathological features of patients with CRC (Table 1). The expression of *TDRKH-AS1* is significantly different in distant metastasis (absent vs. present, *p* = 0.078), tumor size in centimeters (≤5 vs. >5 cm, *p* = 0.019), and depth of tumor in the first groups (T1 + T2 vs. T3 + T4, *p* = 0.038). We further identified that the *TDRKH-AS1* was significantly upregulated in CRC tissues compared with the corresponding adjacent non-tumor tissue (Figure 2A). More than 50% of CRC patients have an upregulated expression of *TDRKH-AS1* in their tumor tissues (Figure 2B). The results showed that the *TDRKH-AS1* expression level was associated with the prognosis of the CRC patients, and its overexpression has clinically related to poor prognosis in patients with CRC (Figure 1B). Furthermore, by measuring the size of tumors in CRC patients, we found that the patients with a higher *TDRKH-AS1* level have a larger tumor size than those patients who have a lower *TDRKH-AS1* level (Figure 2C). Additionally, we observed poor survival outcome of CRC for patients with high expression of *TDRKH-AS1* (Figure 2D) These results implied that the high expression of *TDRKH-AS1* in CRC patients' tumor tissues is an important event, and it could be a prognostic diagnostic indicator in clinical settings.

**Table 1:** Relationships between *TDRKH-AS1* expression and clinical pathological features of patients with colorectal cancer (CRC).

**Parameter**	**No. of patients**	***TDRKH-AS1* (low)**	***TDRKH-AS1* (high)**	***p*-value**
Sex				0.315
male	14	6	8	
female	16	9	7	
Age (years)				0.226
<60	8	3	5	
≥60	22	10	12	
Distant metastasis				0.078
Absent	13	6	7	
Present	17	8	9	
Tumor size (cm)				0.019
≤ 5	11	8	3	
>5	19	4	15	
Depth of tumor				0.038
T1 + T2	7	3	4	
T3 + T4	23	6	17	

### *TDRKH-AS1* Promotes Proliferation and Invasion of CRC Cells

**Figure 3:**
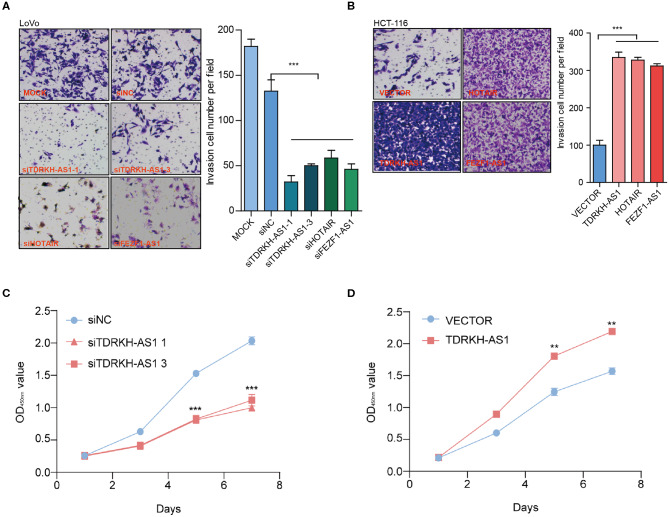
*TDRKH-AS1* increases the proliferation and invasion of colorectal cancer (CRC) cells *in vitro*. **(A)** Through the Transwell assay, knockdown of *TDRKH-AS1* can significantly inhibit the invasion ability of the LoVo cells. **(B)** Overexpression of *TDRKH-AS1* can significantly promote the invasion ability of the HCT-116 cell lines. **(C)** Knockdown of *TDRKH-AS1* significantly inhibited the ability of proliferation of the LoVo cells in the cell counting kit-8 (CCK-8) assay. **(D)** Overexpression of the *TDRKH-AS1* significantly promoted the ability of proliferation in the HCT-116 cell lines. Statistics were considered to be at a significant level when *p* < 0.05, where ^*^p < 0.05, ^**^p < 0.01, and ^***^p < 0.001.

Most of CRC patients died from an exacerbation caused by the proliferation of cancer cells, so the proliferation and invasion of CRC cells play a crucial role in the occurrence and development of CRC. We further tested whether the cell invasion and migration potential were modified as a result of the *TDRKH-AS1* expression level alteration. To prove the effects of *TDRKH-AS1* on CRC cell proliferation and tumorigenicity, we designed five different specific small interfering RNAs (siRNAs), siTDRKH-AS1 1 (siT-1) to siT-5, which were able to knock down the expression of the *TDRKH-AS1* through silencing it. In the Transwell assay, the results showed that interfering with *TDRKH-AS1* expression could inhibit cell invasion in the HCT-116 cell line, and siT-1 and siT-3 had a better knockdown effect (Figure 3A). When we overexpressed the *TDRKH-AS1* in the HCT-116 cell line, the number of invasive cells increased, which indicated that the invasion ability of cells increased significantly (Figure 3B). We further applied the CCK-8 assay in the LoVo cells to examine the proliferative changes after we under- and overexpressed the *TDRKH-AS1*. We found that the LoVo cell proliferation was consistent with the regulation of the *TDRKH-AS1*. The reduction was viewed after we underexpressed the *TDRKH-AS1* (Figure 3C), and the promotion of cell proliferation was viewed after we overexpressed the *TDRKH-AS1* (Figure 3D). These results demonstrated that the expression of *TDRKH-AS1* has a positive correlation with cellular invasion and proliferation.

### *TDRKH-AS1* Performs Its Biological Functions Through the *Wnt* Signaling Pathway

**Figure 4:**
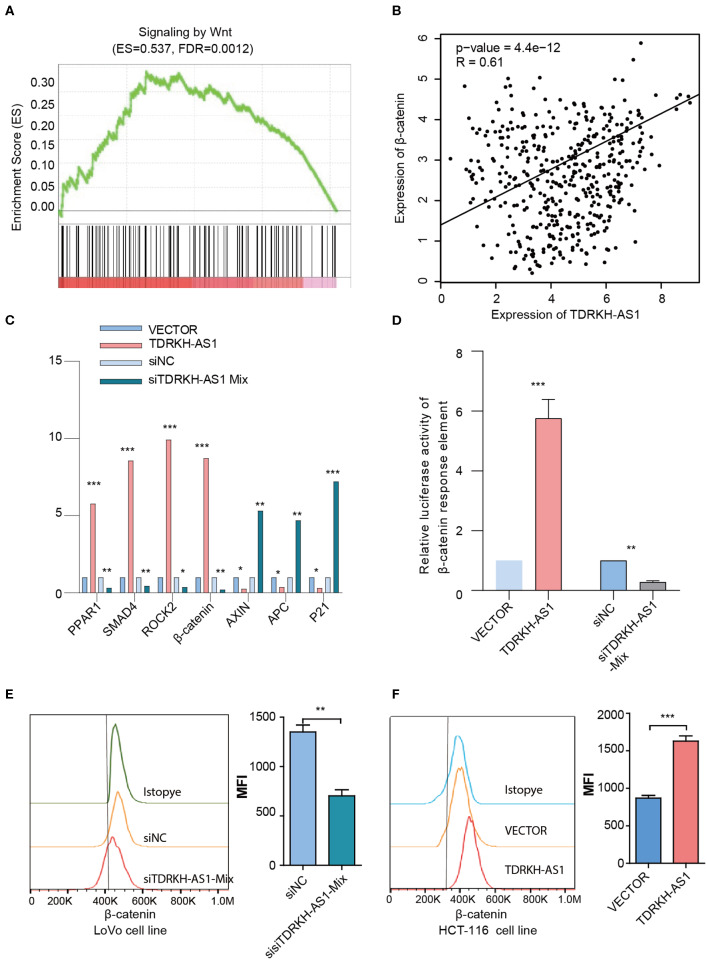
*TDRKH-AS1* performs its biological functions through the Wnt signaling pathway. **(A)** The gene set enrichment analysis of the *Wnt* signaling pathway. **(B)** The expression of *TDRKH-AS1* was significantly positively correlated with the expression of β-catenin. **(C)** The quantitative real-time PCR results show that overexpression or knockdown of the *TDRKH-AS1* significantly affected the expression of driver genes in the *Wnt* signal pathway. **(D)** The luciferase activity shows that overexpression or knockdown of *TDRKH-AS1* will significantly affect the expression of β-catenin. **(E,F)** The flow cytometry assay shows that overexpression or knockdown of the expression of *TDRKH-AS1* can affect the expression of the β-catenin protein. Statistics were considered to be at a significant level when *p* < 0.05, where ^*^p < 0.05, ^**^p < 0.01, and ^***^p < 0.001.

The survival and prognosis of CRC remain relatively poor since the recurrence and metastasis. Therefore, it is imperative to understand the molecular pathogenesis of CRC in order to develop effective treatment strategies to improve the prognosis of patients. To investigate the mechanism, we used the expression data from CRC samples in the TCGA database to analyze lncRNA coexpressed genes by the method of GSEA. The coexpressed genes with *TDRKH-AS1* were enriched in the *Wnt* signaling pathway (enrichment score = 0.537, FDR = 0.0012) in CRC from GSEA (Figure 4A). Further enrichment analysis of *TDRKH-AS1* and β-catenin, a core protein in the *Wnt* signaling pathway, using the data in TCGA showed that the expression of this lncRNA was significantly positively correlated with the expression of β-catenin (Figure 4B) (14). After overexpression or knockdown of the expression of *TDRKH-AS1*, we conducted an expression analysis of driver genes in the *Wnt* signaling pathway and found that overexpression or knockdown of the expression of this lncRNA could significantly affect the expression of driver genes in the *Wnt* signaling pathway. The quantitative real-time PCR (qRT-PCR) assay indicated that *TDRKH-AS1* influenced the *Wnt* signaling pathway at the RNA level (Figure 4C). The oncogenes, including the *PPAR1, SMAD4, ROCK3*, and β-catenin, are significantly increased, and the tumor suppressor genes, including the *AXIN, APC*, and *P21*, are significantly decreased when the cells were transfected with *TDRKH-AS1*. According to the luciferase activity assay, we identified that the knockdown of the expression of *TDRKH-AS1* could reduce the luciferase activity of β-catenin and vice versa (Figure 4D). Furthermore, at the protein level, through the flow cytometry assay, we found that the expression of *TDRKH-AS1* was positively correlated with the expression of β-catenin (Figures 4E,F).

## Discussions

LncRNAs play essential roles in human cancer progression, especially in cholangiocarcinoma (10) and CRC (25). Recently, lncRNAs are popular to be studied since it is a biomarker for us to pursue better cancer detection and treatment with a non-invasive procedure (25). The associations between the CRC and multiple lncRNAs were reported from previous studies, such as *MIR22HG* (26), *FEZF1-AS1* (17), and *LINC00152* (27). However, *TDRKH-AS1* was only reported from a computational approach from the TCGA dataset in lung adenocarcinoma (13). Our study first elucidated the *TDRKH-AS1*, a lncRNA that significantly influences the occurrence and development of CRC through the *Wnt* signaling pathway. It could affect the expression of driver genes of the *Wnt* signaling pathway, improves the luciferase activity of β-catenin, and promotes the expression of β-catenin at the protein level. In our research, we found that *TDRKH-AS1* was overexpressed in 53% of CRC patients, and its expression was positively correlated with patient prognosis and tumor size. It enhances the ability of colon cancer cells to proliferate and metastasize.

Generally, the expression of the lncRNA is relatively low, but we located the *TDRKH-AS1* based on the TCGA CRC cohort, the largest free public database integrated multiomics' and clinical data for all types of cancers. Specifically, the RNA-sequencing (RNAseq) data from TCGA focused on exon sequences (28). Further population-scale deep whole-genome sequencing would help to detect the signal more accurate and robust (29). In our experiment, we emphasized the differential gene expressions between patients in different stages. The comparison between the healthy population and the CRC patients may provide more strong signals. Furthermore, including the *TDRKH-AS1*, we found more targets through the RNAseq analysis, which may have potential effects on the cancer progress. Further analyses on the other identified genes might help to find new targets for better understanding the CRC.

In our study, we also observed the fluctuations of tumor suppressor genes and oncogenes in the RNA level, while *TDRKH-AS1* was introduced into the cells. We showed that *TDRKH-AS1* promotes cancer proliferation through the *Wnt* signaling pathway in CRC patients. However, the specific target for *TDRKH-AS1* in the *Wnt* signaling pathway is still unclear. The mechanism of *TDRKH-AS1* in regulating genes could be investigated to clarify its function in later *in vitro* and *in vivo* experiments.

In conclusion, *TDRKH-AS1* plays its carcinogenic biological function by activating the *Wnt* signaling pathway in colorectal cancer, and its expression is significantly positively correlated with the prognosis of patients. Currently, many studies provided the potential inhibitors of the *Wnt* signaling pathway. The major inhibitors for the *Wnt* signal pathway include Dickkopf-1 (DKK-1) (30, 31), Frizzled-related protein (sFRP) (32–34), Wnt inhibitory factor-1 (WIF-1) (31, 35, 36), Ceberus (CER) (31), and salinomycin (37). These potential inhibitors for targeting the *TDRKH-AS1* is worth to be constructed and evaluated in future studies. It is also feasible and vital to find new therapeutic targets in the *Wnt* pathway by inhibiting the proliferation of tumor cells.

## Data Availability Statement

The datasets generated for this study can be found in The Cancer Genome Atlas (TCGA) in CRC subgroup. This data can be found here: Carlson (24). org.Hs.eg.db was used for genome wide annotation for human.

## Ethics Statement

The studies involving human participants were reviewed and approved by The Affiliated Hospital of Jiangnan University. The patients/participants provided their written informed consent to participate in this study. TCGA Ethics and Policies was originally published by the National Cancer Institute.

## Author Contributions

JwZ and JyZ conceived and performed the experiments and wrote the manuscript. MS collected the patient samples and interpreted the data. YaJ, JlZ, YeJ, YY, and LZ analyzed the experimental data. JyZ performed the RNA-seq data analyses and visulizations. All authors read and approved the final draft of the manuscript.

## Conflict of Interest

The authors declare that the research was conducted in the absence of any commercial or financial relationships that could be construed as a potential conflict of interest.

## Supplementary Material

Supplement 1 Distribution of age in different cancer stages with **(A)** gender, **(B)** vitual status, **(C)** disease type, and **(D)** primary site.

Supplement 2 Distribution of the fold change for genes between the different stages.

Supplement 3 Gene enrichment analysis by referencing the biological processes between the stage IIIC and stage I for CRC patients.

Supplement 4 Gene enrichment analysis by referencing the molecular functions between the stage IIIC and stage I for CRC patients.

Supplement 5 Gene enrichment analysis by referencing the cellular component between the stage IIIC and stage I for CRC patients.
