# Supplementary figures and images for: Long Non-coding RNA TDRKH-AS1 Promotes Colorectal Cancer Cell Proliferation and Invasion Through the β-Catenin Activated Wnt Signaling Pathway
Source: Front Oncol. 2020 May 15;10:639. doi: 10.3389/fonc.2020.00639 (PMC7326065; doi:10.3389/fonc.2020.00639)

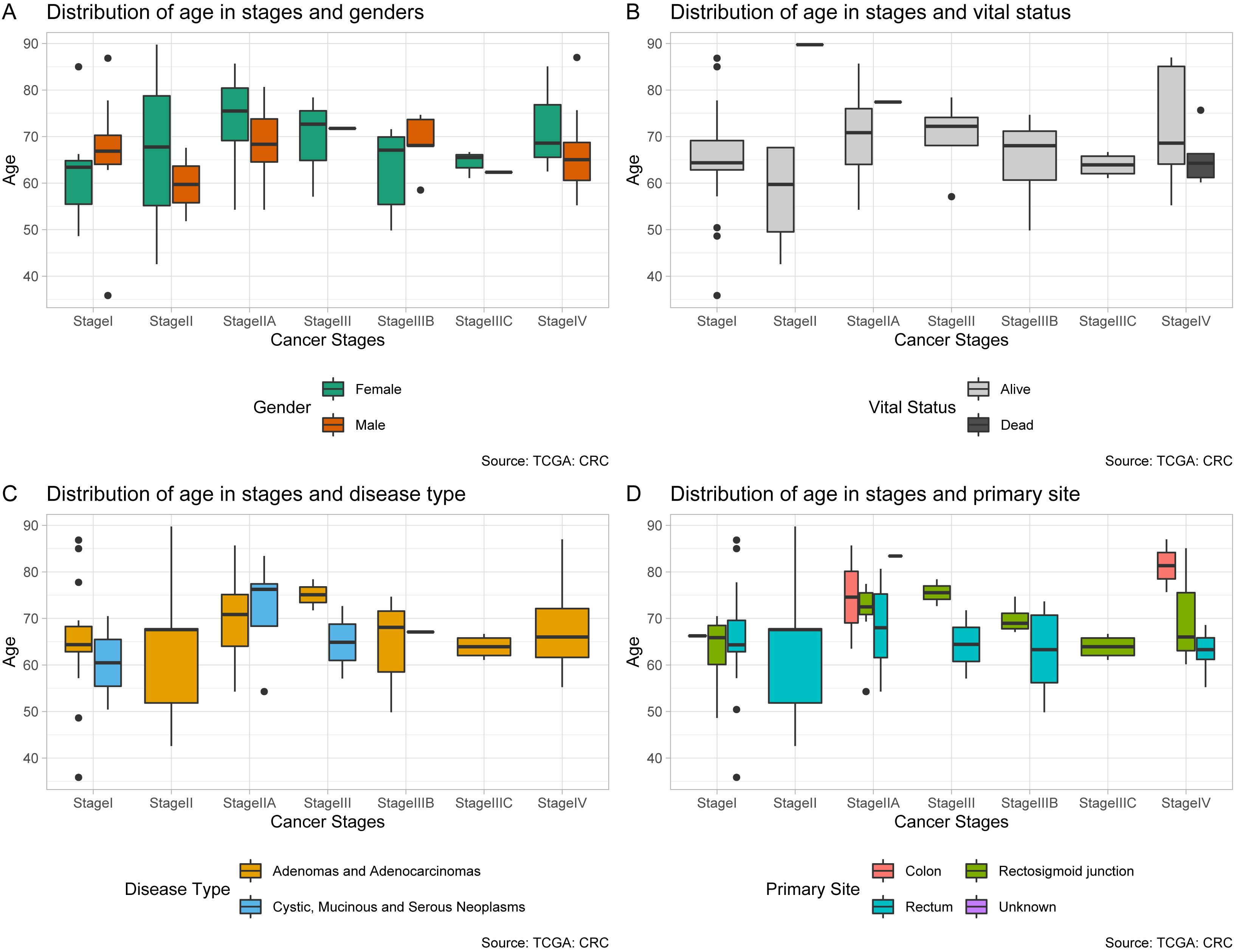

Supplement: Supplement 1 — Distribution of age in different cancer stages with (A) gender, (B) vitual status, (C) disease type, and (D) primary site. [file Image_1.TIF]

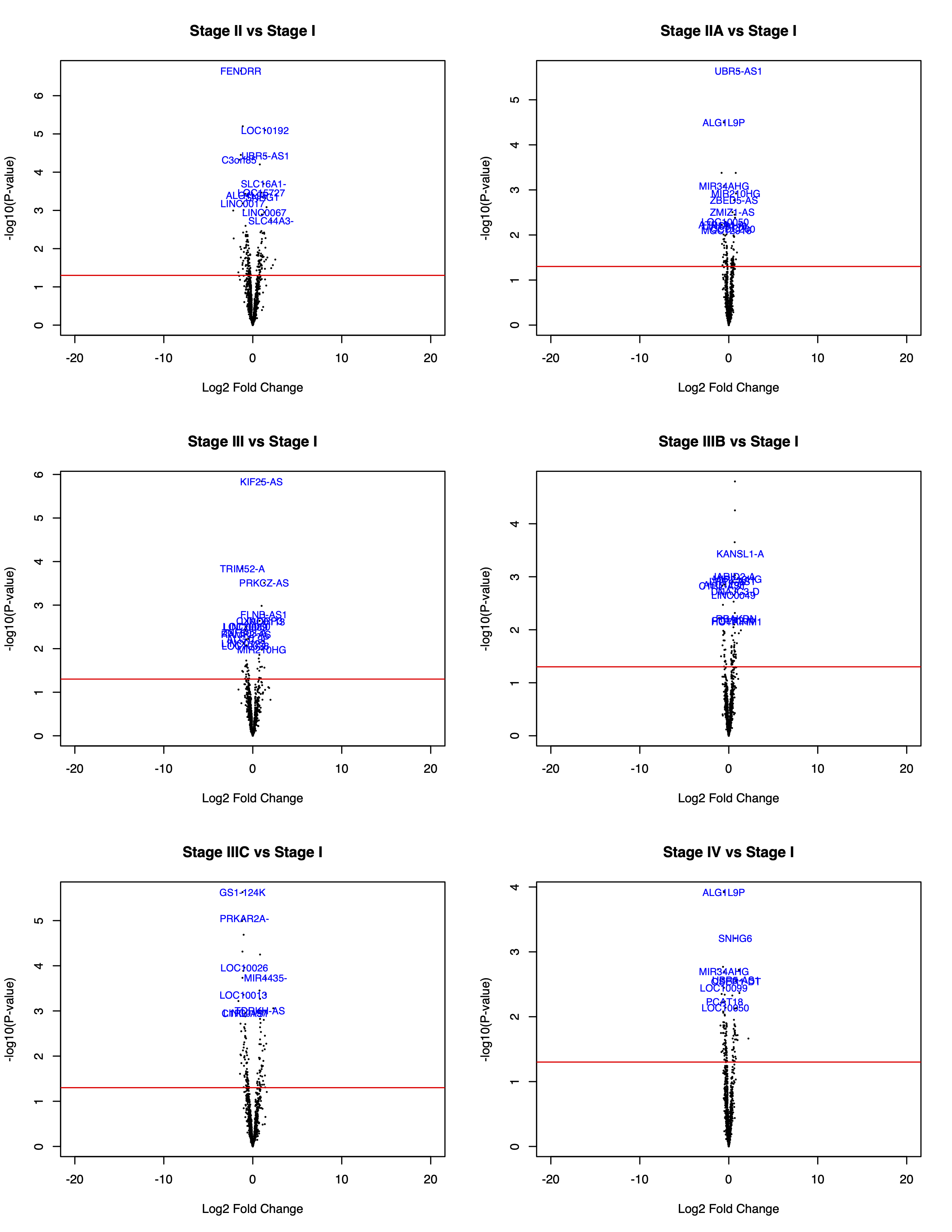

Supplement: Supplement 2 — Distribution of the fold change for genes between the different stages. [file Image_2.TIF]

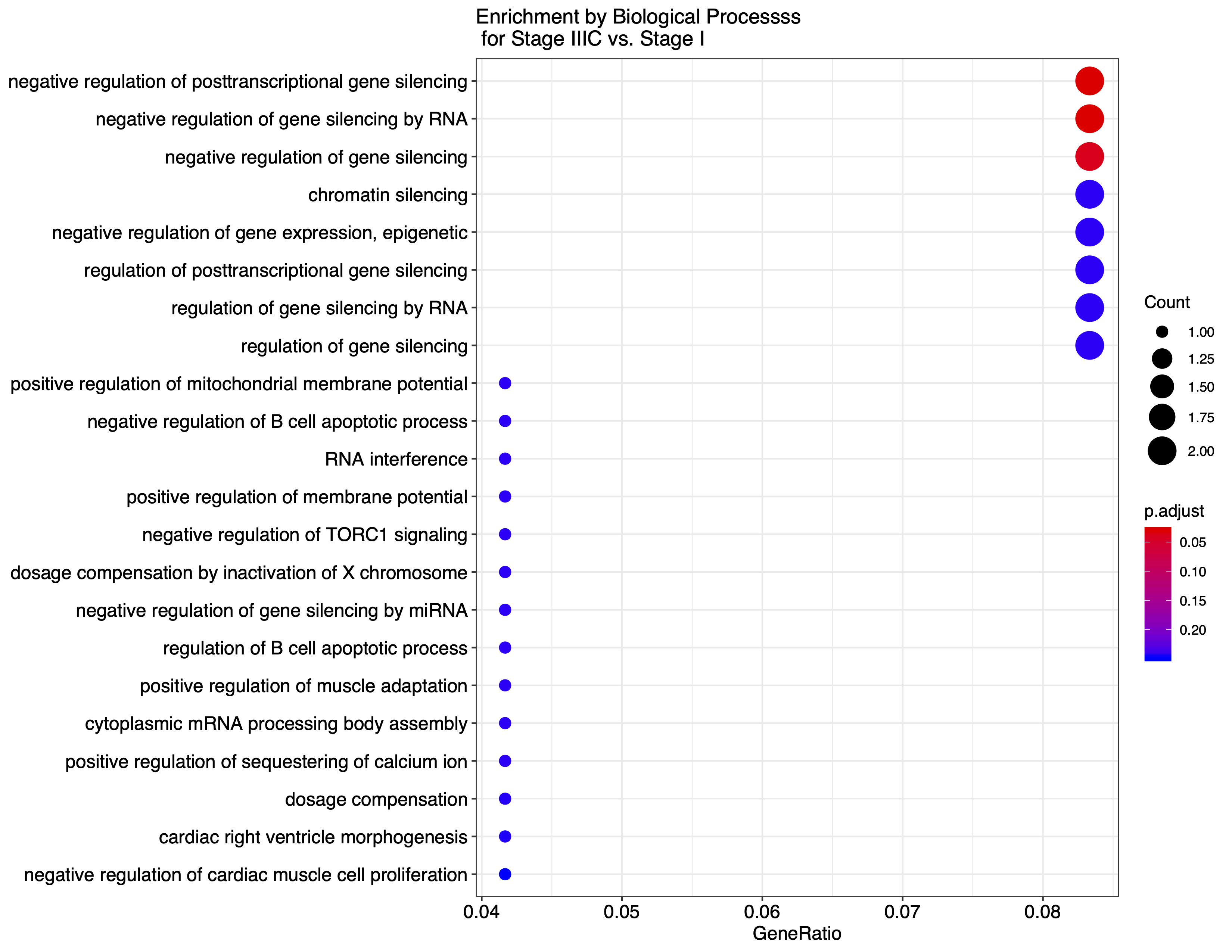

Supplement: Supplement 3 — Gene enrichment analysis by referencing the biological processes between the stage IIIC and stage I for CRC patients. [file Image_3.TIF]

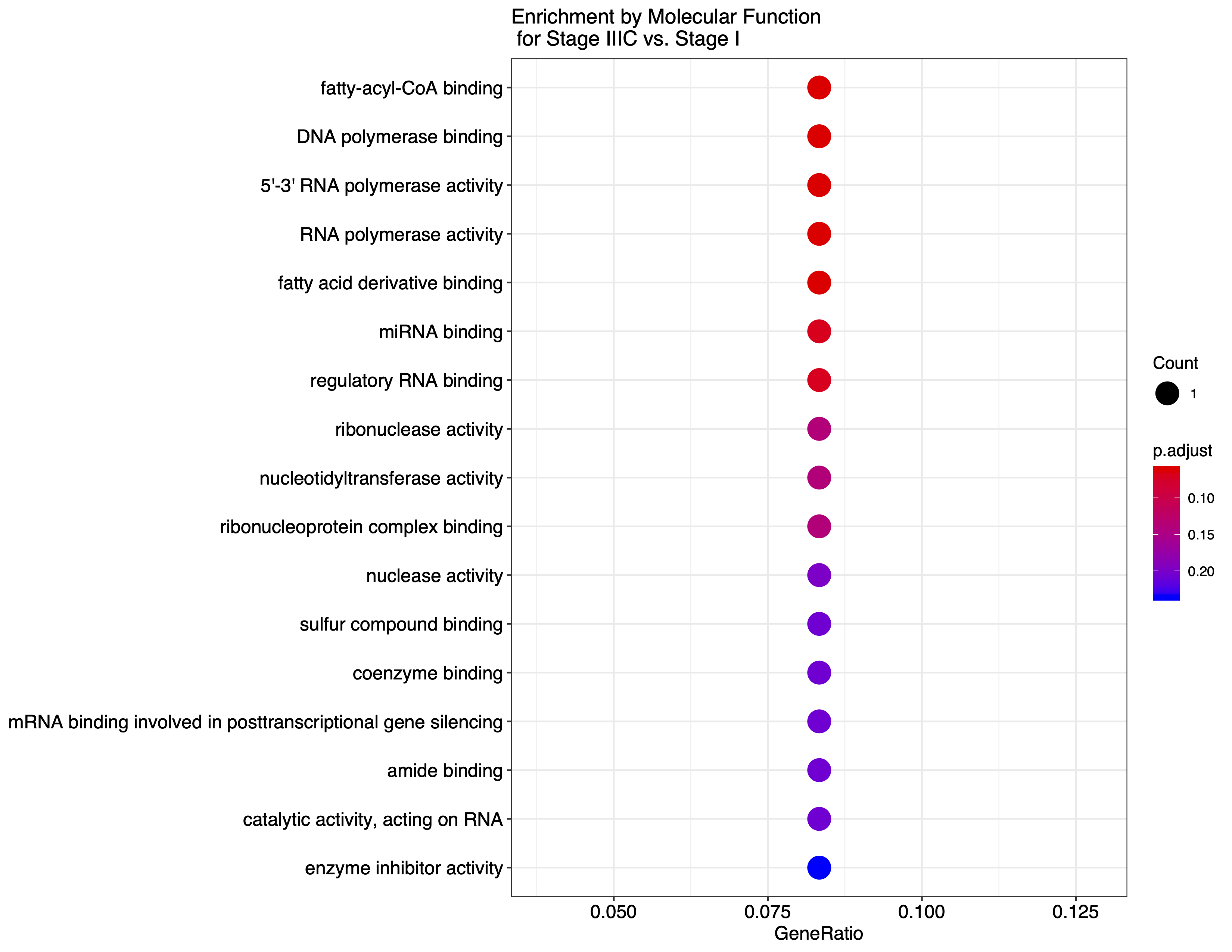

Supplement: Supplement 4 — Gene enrichment analysis by referencing the molecular functions between the stage IIIC and stage I for CRC patients. [file Image_4.TIF]

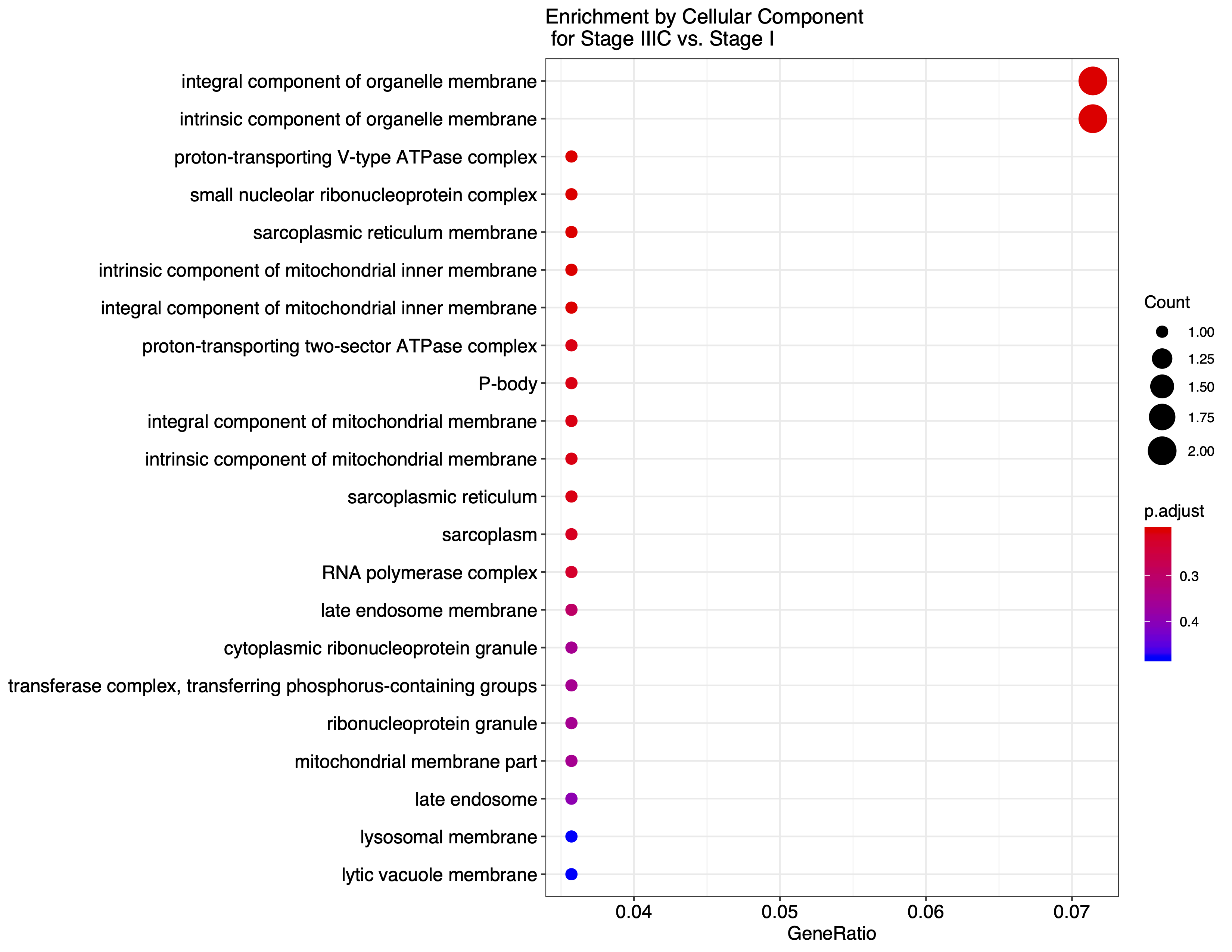

Supplement: Supplement 5 — Gene enrichment analysis by referencing the cellular component between the stage IIIC and stage I for CRC patients. [file Image_5.TIF]
